# Promising results of surgical management of advanced medication related osteonecrosis of the jaws using adjunctive leukocyte and platelet rich fibrin

**DOI:** 10.1186/s12903-021-01965-7

**Published:** 2021-12-01

**Authors:** Öznur Özalp, Nelli Yıldırımyan, Canan Öztürk, Burak Kocabalkan, Göksel Şimşek Kaya, Alper Sindel, Mehmet Ali Altay

**Affiliations:** 1 Department of Oral and Maxillofacial Surgery, Faculty of Dentistry, Akdeniz University, Dumlupinar Boulevard, Campus, 07058 Antalya, Turkey; 2 Private Practice, Istanbul, Turkey; 3 Antalya Training And Research Hospital, Antalya, Turkey; 4 Topraklik Centre of Oral and Dental Health, Ankara, Turkey

**Keywords:** Medication-related osteonecrosis of the jaw, Osteonecrosis, Surgical treatment, Adjunctive therapy, Leukocyte and platelet rich fibrin, Platelet concentrates

## Abstract

**Background:**

Leukocyte- and platelet-rich fibrin (L-PRF) is an autologous matrix scaffold which regulates inflammation by stimulating cytokines and growth factors that are involved in the immune response. L-PRF is suggested as a viable adjunctive method to surgical interventions due to its advantages on tissue healing. This study aims to retrospectively evaluate the adjunctive role of L-PRF in surgically treated medication-related osteonecrosis of the jaws (MRONJ) patients.

**Methods:**

Between January 2012 and December 2020, patients with AAOMS stage II and III MRONJ lesions, who were treated surgically with adjunctive use of L-PRF in the authors’ institution were enrolled. Surgical interventions consisted of either marginal resection or sequestrectomy with peripheral ostectomy (SPO) or curettage and L-PRF application. Medical records of these patients were retrospectively reviewed and healing was assessed according to certain parameters including mucosal closure and presence of infection, exposed bone, fistula or radiologic markers of disease progression for a minimum of 12 months.

**Results:**

Thirteen patients (7 women and 6 men) with an average age of 72.4 years (± 10.61, range 54–84) were included in the study, nine of whom had AAOMS stage III and four stage II MRONJ. Three patients had a marginal resection, nine patients had sequestrectomy with peripheral ostectomy (SPO) and one patient underwent a curettage procedure. All marginal resection and six SPO patients showed complete healing while four patients, who had SPO or curettage experienced incomplete healing. Mean follow up was 20.1 ± 18.29 months.

**Conclusion:**

The use of L-PRF may be a favorable adjunctive option in the treatment of MRONJ owing to its favorable effects on tissue repair, ease of application, minimally invasive and cost-effective character and autogenous nature.

*Trial registration* Retrospectively registered.

## Background

In 2003, Robert Marx has introduced and defined the term “Bisphosphonate (BP) Related Osteonecrosis of the Jaws” (BRONJ) as an adverse effect of pamidronate and zoledronate [1]. Recent studies have revealed denosumab (a RANK ligand inhibitor), bevacizumab, sunitinib and several other antiangiogenic agents as other potential medications in the etiology of osteonecrosis [2, 3]. Consequently in 2014, the American Association of Oral and Maxillofacial Surgeons (AAOMS) has revised the nomenclature and recommended the use of the term “medication-related osteonecrosis of the jaw” (MRONJ) [4].

The diagnostic criteria for MRONJ include exposed bone or fistula that probes to the bone in the maxillofacial region that has not healed within eight weeks of identification, current or previous exposure of an antiresorptive or antiangiogenic medication and absence of previous radiotherapy to the maxillofacial region or obvious metastatic disease to the jaws [4]. MRONJ may occur spontaneously or it may be induced by local trauma, such as extractions or ill-fitting dentures, or oral surgical procedures [1].

Changes in bone remodeling, inflammation or infection, immune deterioration, inhibition of angiogenesis or soft tissue toxicity due to medications have been previously proposed among pathological mechanisms responsible for osteonecrosis; although the true pathophysiology remains to be debated [5, 6]. This limited understanding of the pathophysiology of MRONJ has so far resulted in a failure to reach a globally accepted consensus on its prevention and treatment.

For asymptomatic MRONJ lesions, AAOMS recommends conservative methods using antibacterial mouth rinses, analgesics or systemic antibiotics to keep pain and infection under control [4]. Conservative approaches have been suggested for all MRONJ patients, regardless of the stage of the disease, from the diagnosis onward; but the unsatisfactory results of conservative treatment in AAOMS stage 2 and 3 MRONJ patients suggest that surgical approaches may be more preferable in advanced stages [4, 7, 8].

In addition to medical and surgical approaches, several adjunctive treatment methods have also been proposed for the management of MRONJ. Among these measures are low level laser therapy, hyperbaric oxygen, ozone therapy, and treatments using teriparatide, bone morphogenic protein-2 (BMP-2), pentoxyphilline, alpha-tocopherol or autologous platelet concentrates (APCs); each of which has conflicting success rates [9–19].

Leukocyte- and platelet-rich fibrin (L-PRF) is one of the APCs described by Choukroun et al. [20]. It is described as an autologous matrix scaffold, which regulates inflammation by stimulating cytokines and growth factors that are involved in the immune response [20]. It has been reported that the mechanism of L-PRF is based on the secretion of essential growth factors and proteins including Vascular Endothelial Growth Factor (VEGF), Transforming Growth Factor-β1 (TGF-β1), Bone Morphogenetic Proteins (BMPs), Transforming Growth Factor-β2 (TGF-β2),-a fundamental factor in bone healing-, Insulin–like Growth Factor (IGF), and Platelet-Derived Growth Factor (PDGF) by the activation of platelets [21]. L-PRF is suggested as a viable adjunctive method to surgical interventions due to its advantages on tissue healing. Therefore, this study aims to retrospectively evaluate the adjunctive role of L-PRF in surgically treated MRONJ patients.

## Methods

### Study design and data collection

**Fig. 1:**
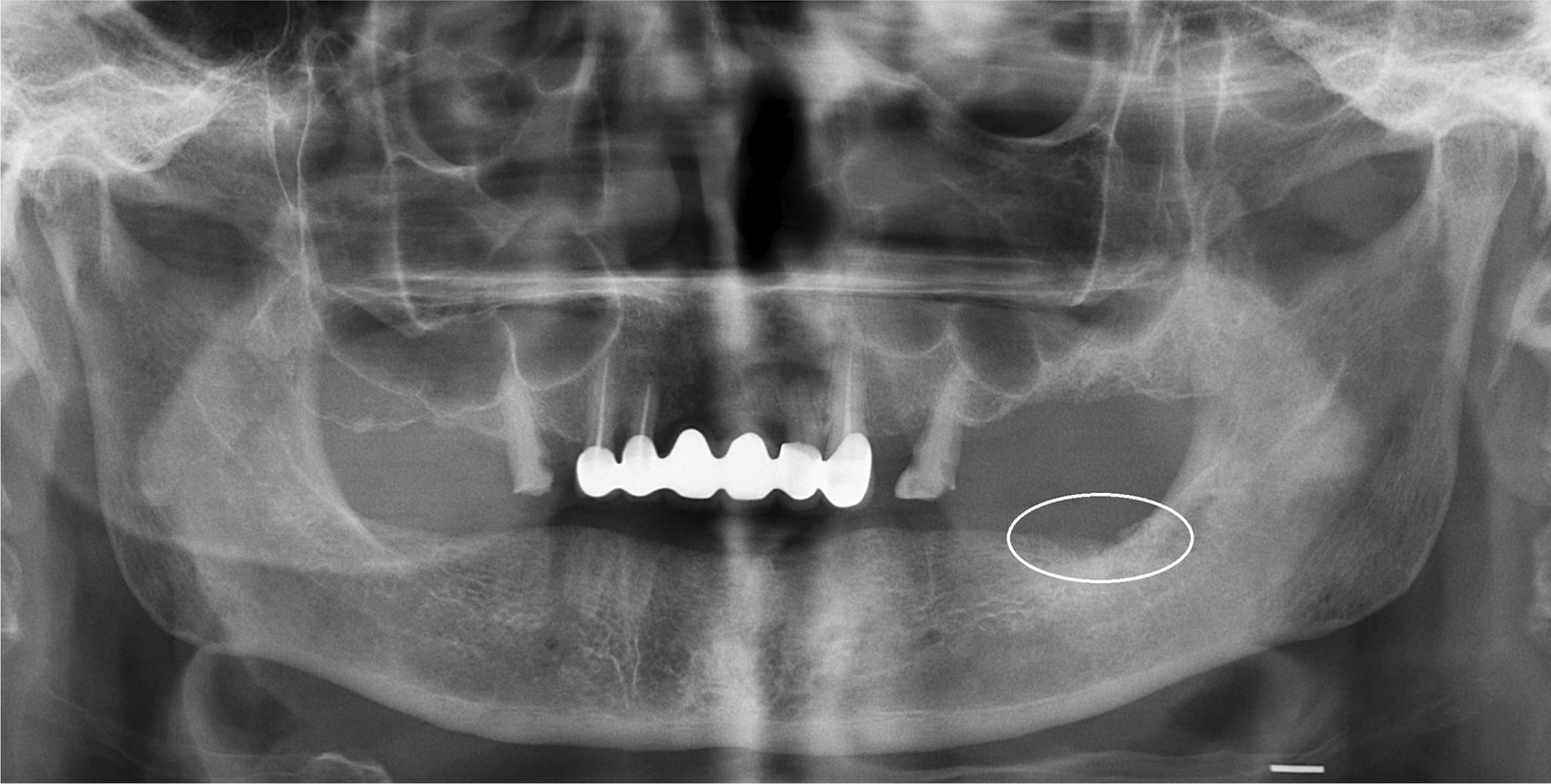
Preoperative panoramic radiograph of a patient with AAOMS Stage II MRONJ lesion on the left, posterior mandible. Encircled area shows the area of osteonecrosis

Patients with AAOMS stage II and III MRONJ lesions, who were treated surgically with adjunctive use of L-PRF between 2012 and 2020 at the Department of Oral and Maxillofacial Surgery in the authors’ institution were evaluated. Details of medical history were recorded for each patient and MRONJ was confirmed after clinical and radiologic examinations (Fig. 1).

Patients with a history of radiotherapy to the head and neck region were excluded. A database was created including gender, age, primary disease, type and route of medication, and treatment details. An informed consent was obtained from each patient before any surgical intervention. This study was conducted in accordance with the Declaration of Helsinki on medical protocol and was approved by the University’s Ethical Review Board with the approval number of 664/26.09.2018.

### Acute phase intervention

Patients with acute symptoms were prescribed antibiotics (amoxicillin/clavulonate 875 mg/125 mg, twice daily or clindamycin 150 mg, four times daily in patients with known drug allergies, and an antibacterial mouth rinse (0.12% CHX). Additionally, peripheries of the necrotic lesions were irrigated with sterile saline solution and rifamycine until the acute phase subsided. Patients were consulted with their primary physicians for drug discontinuation. A drug holiday was initiated from MRONJ-diagnosis onward and at least six months before any surgical intervention.

### Surgical procedure and PRF preparation

**Fig. 2:**
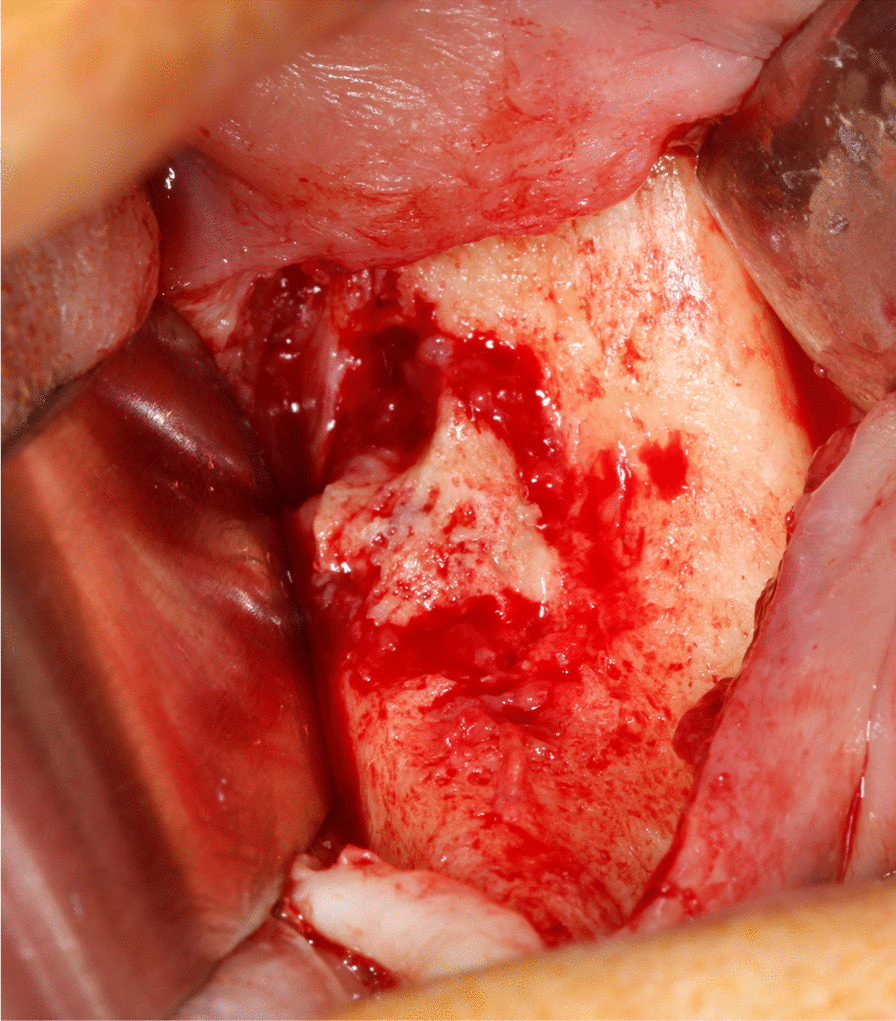
Intraoperative view of the lesion

An oral antibiotic (using the same regimen mentioned above) was administered 2 days pre-operatively. All patients except one (under general anesthesia) were operated under local anesthesia. The surgical intervention consisted of either marginal resection or sequestrectomy with peripheral ostectomy (SPO), followed by irrigation, L-PRF application and tension-free primary closure. The decision regarding the choice of surgical intervention was made intraoperatively according to the surgeon's assessment of the local extent of the disease (Fig. 2).

**Fig. 3:**
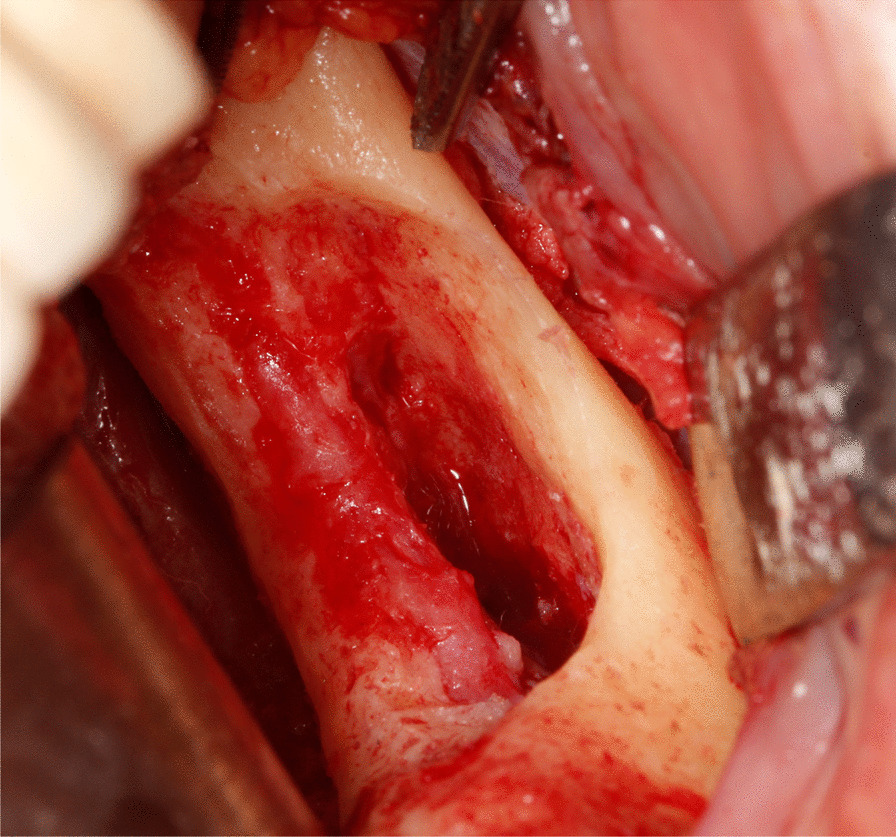
Intraoperative view of the defect following removal of the lesion and peripheral ostectomy

Sequestra were removed using surgical curettes. Peripheral ostectomy and removal of sharp bone margins were performed using rotary instruments. Ostectomy was continued until the apparently healthy, bleeding bone was reached. Debris was removed under copious irrigation with sterile saline solution. Afterwards, rifamycine was applied to the surgical area (Fig. 3).

**Fig. 4:**
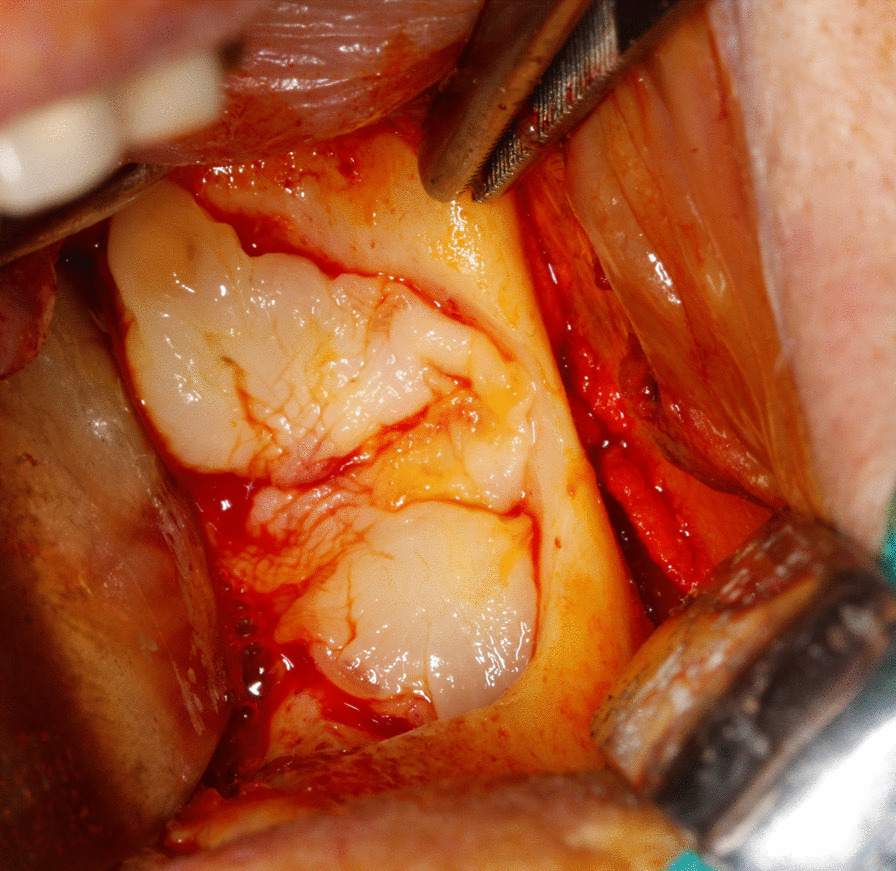
L-PRF application to the residual bone defect

**Fig. 5:**
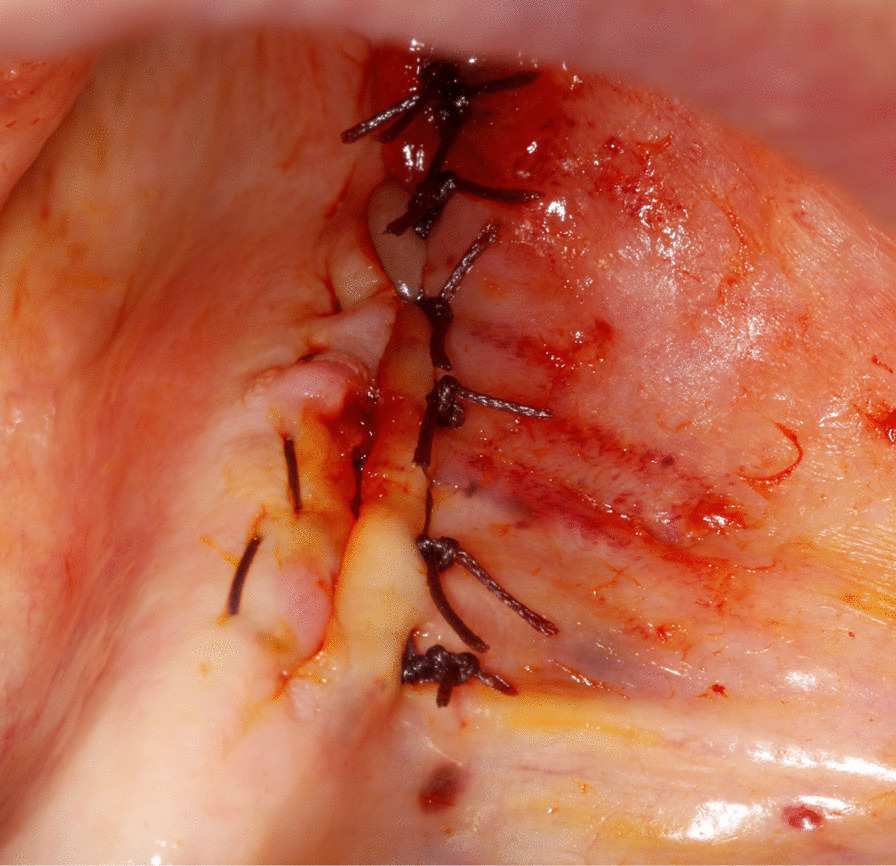
Tension-free primary closure of the wound site

Intraoperatively, peripheral blood was collected and centrifuged in tubes without anticoagulant agents at 2700 rounds per minute for 12 min. Residual bone defect was filled and covered with L-PRF membrane and the flap was primarily closed in a tension‐free manner (Figs. 4, 5).

Resected tissues were examined histopathologically to exclude the possibility of an unsuspected malignancy.

### Postoperative management and success criteria

**Fig. 6:**
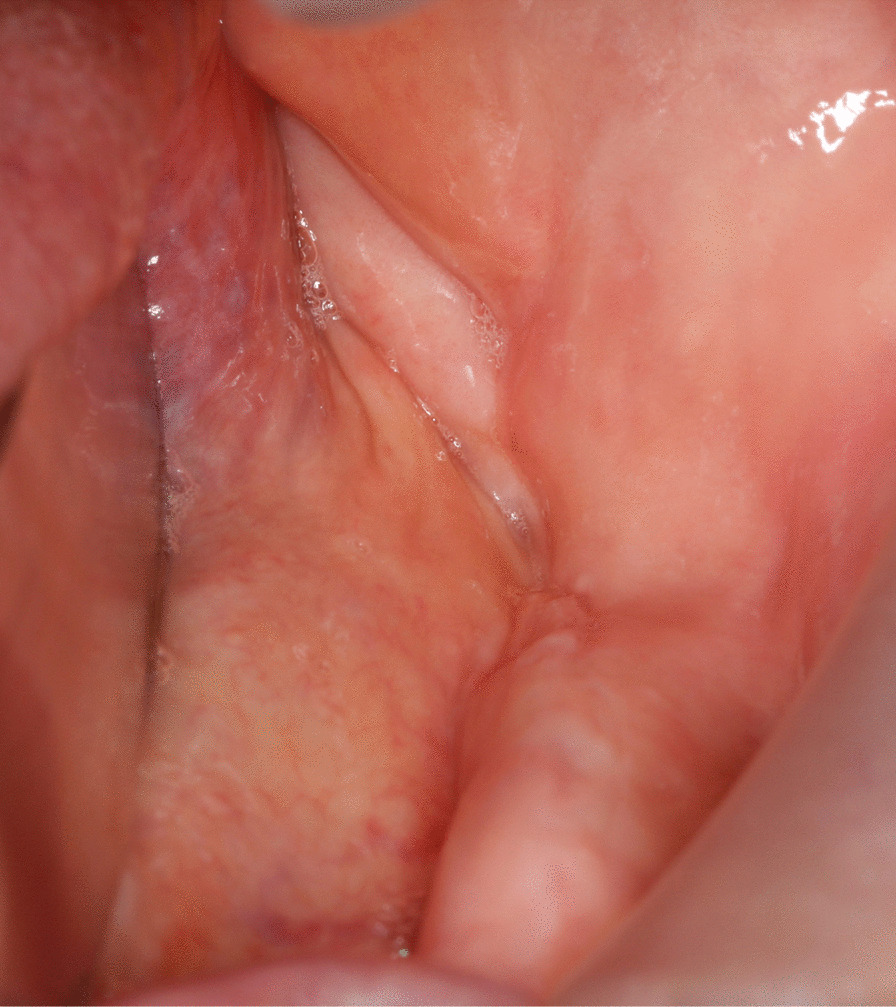
Post-operative, intraoral view of the patient showing complete healing at 6 months after surgery

**Fig. 7:**
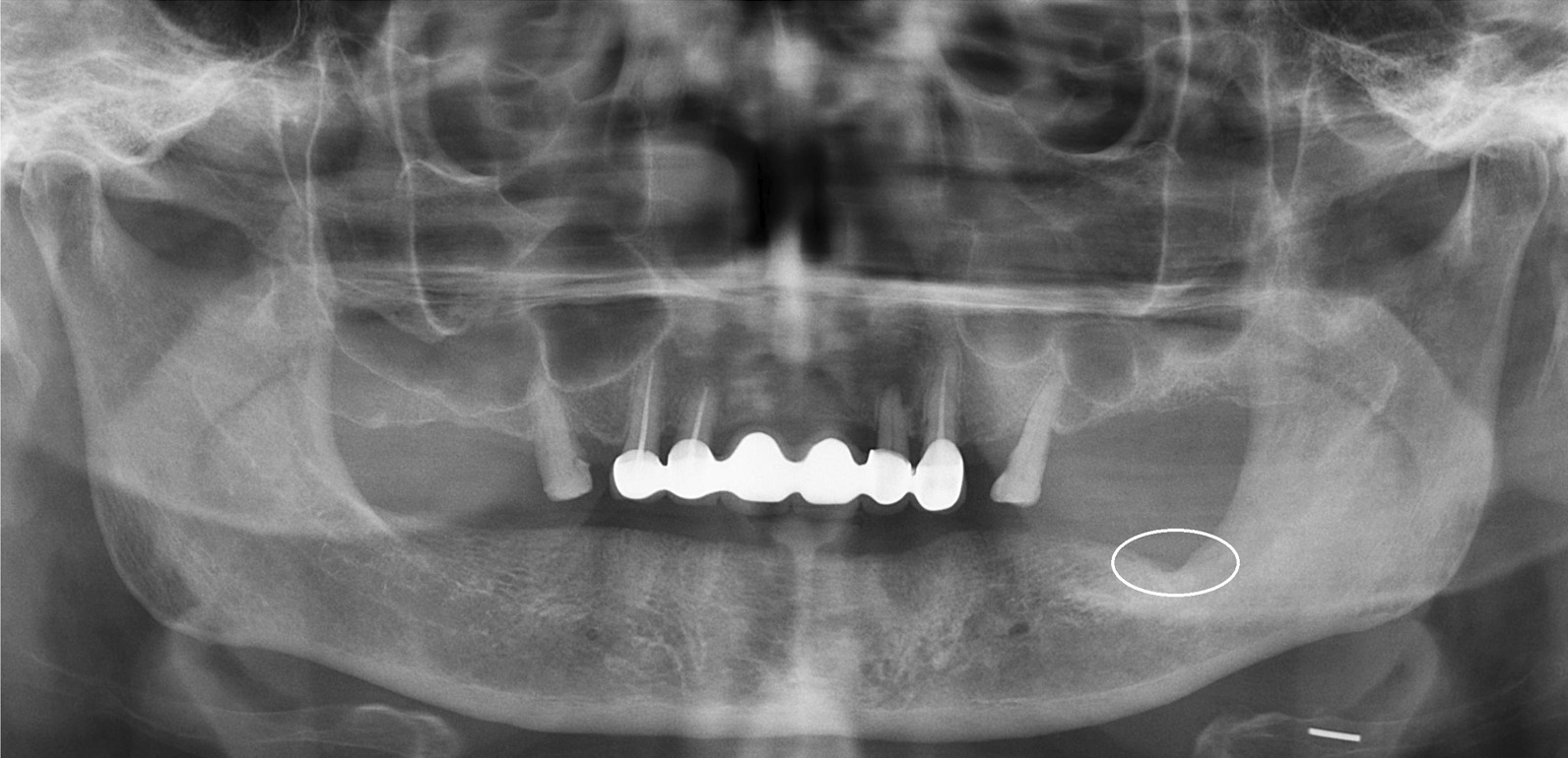
Postoperative panoramic radiograph of the patient showing arrest of disease progression and remodeling of the affected area at six months after surgery. Encircled area shows no sign of osteonecrosis or new sequestrum

Systemic antibiotics were continued twice daily for one week. Surgical area was irrigated with sterile saline solution every other day. Sutures were removed after 10 days. All patients were followed up weekly for a month and quarterly afterwards for a year. Treatment success was determined according to clinical and radiographic outcomes, as previously proposed by Silva et al. [22] Patients with intact mucosal closure and no signs of infection, exposed bone, fistula or radiologic markers of disease progression for the post-operative 12 months were considered “completely healed” [22] (Figs. 6, 7). Failure to fulfill any of these criteria was considered as an “incomplete healing”.

## Results

Thirteen patients (7 women and 6 men) with an average age of 72.4 years (± 10.61, range 54–84) met the inclusion criteria, nine of whom had AAOMS stage III and four stage II MRONJ. Eleven patients had a history of intravenous BP (zoledronate) therapy and one patient was treated with an oral BP (alendronate). One other patient was initially treated with oral BP (ibandronate), was then given intravenous BP (zoledronate) and finally received denosumab. No other antiangiogenic or antiresorptive drugs were reported. These patients had been prescribed BPs due to prostate cancer, breast cancer, multiple myeloma or osteoporosis.

**Fig. 8:**
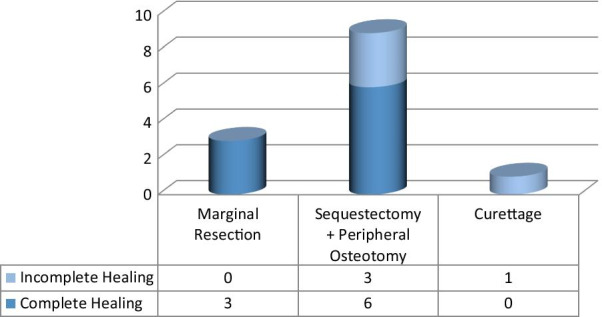
Treatment outcomes with respect to surgical interventions

Three patients had a marginal resection, nine patients had SPO and one patient underwent a curettage procedure. All marginal resection and six SPO patients showed complete healing while three patients, who had SPO experienced incomplete healing (Fig. 8).

The latter were re-operated; however, one experienced a persistent oro-antral fistula, which healed partially after two oral mucosal flap advancement surgeries, at 22 months of follow up. The other patient with incomplete healing suffered from continuous bone exposures despite four consecutive surgeries. Infection, however, was eliminated and subjective symptoms including pain and discomfort improved in both cases with incomplete healing. The third patient was re-operated for sequestrectomy and healed completely after this second intervention. Finally, the patient who underwent a curettage procedure, also showed complete resolution after the chronic fistula and pain were eliminated after the third intervention. Detailed data on patient demographics and therapeutic interventions are listed in Table 1. Mean follow up was 20.1 ± 18.3 months.

## Discussion

MRONJ is an adverse reaction of antiresorptive or antiangiogenic medications, the ideal management of which is still a matter of much debate [1, 4]. In addition to the limited knowledge of its pathophysiology, the management of MRONJ is further complicated by various therapeutic options previously reported in the literature. Due to conflicting outcomes of studies reporting on these options, their implications remain merely suggestions and recommendations that are not supported by a high-level of evidence.

Before the change in the nomenclature in 2014, a systematic review evaluated different therapeutic options for the management of BRONJ and reported more than 80% success rate with either marginal or segmental resections and 75% success rate for conservative surgical treatments. These authors have also reported that the success rate declined to 33% using nonsurgical approaches [8]. Although a current review of the literature reveals more favorable outcomes of surgical methods compared to conservative therapies, the updated position paper by AAOMS recommended the use of surgical treatment options in more advanced cases and in those that do not respond to conservative options [4].

Surgical management of MRONJ has been advocated by several authors, who identified surgical resection as an independent predictor of more favorable outcomes as it eliminates the necrotic bone that is unable to regenerate and instead when left untreated, increases the risk of further complications [8, 23, 24]. In 2017, systematic reviews by Comas-Calonge et al. and El-Rabbany et al. both reported better outcomes and higher odds of MRONJ resolution with surgical treatments compared to non-surgical methods [25, 26]. In line with these findings, this study also confirmed surgical therapy as an effective option for patients with advanced MRONJ.

Drug holiday, an approach that still remains controversial in the management of MRONJ, is routinely applied in the authors’ institution, if and when considered medically appropriate by the attending physician(s). Accordingly, bisphosphonate therapy had been terminated before surgical interventions for all patients in this study. The authors of the study believe that this approach may have -to some extent- positively affected outcomes, although the role of drug holiday remains to be debated as bisphosphonates remain active in bones for years after they are discontinued.

In addition to the lack of high-level evidence indicating an ideal therapeutic option, there is also not enough data in the current literature to draw firm conclusions on the efficacy of adjunctive therapies in MRONJ management. Therefore, it was the aim of the present study to evaluate the role of L-PRF, a more recent adjunctive option, in the surgical management of advanced MRONJ. Being autologous and biocompatible materials, APCs were initially suggested as adjunctive methods owing to their positive effects on wound healing, activation of growth factors, stimulation of angiogenesis and local regeneration [15]. Recent reviews investigating efficacy of APCs have concluded that they may be promising adjunctive options for MRONJ management when combined with surgical interventions [27]. Among APCs previously described in the literature, L-PRF was reported to be more stable with an increased lifespan, which allowed the release of cytokines and growth factors over a prolonged time and thus accelerating healing and reducing the risk of postoperative complications [20, 27]. L-PRF was also defined as an efficient osteoconductive and osteoinductive agent due to its promoting effects on osteoblast differentiation and bone maturation [28]. The leukocyte component of L-PRF was shown to provide anti-infective and immune-regulating properties. Additionally, the fibrin architecture of L-PRF is physiologically more stable than other APCs, which enables substantial embedding of growth factors, cytokines, leukocytes, platelets and circulating stem cells into its fibrin network [15, 27]. In a study by Zumstein et al., it was reported that L-PRF provided continuous slow release of growth factors including TGF-β1, VEGF, IGF1 and PDGF for up to 28 days [29]. Furthermore, in a systematic review, L-PRF was shown to be effective on regeneration of bone defects in various oral and maxillofacial interventions [30]. Although the literature provides only limited data on the use of L-PRF for MRONJ patients, our current knowledge indicates that L-PRF is a promising alternative in the treatment of MRONJ [15, 27, 31, 32]. Kim et al. achieved either complete or partial resolution in 95% of MRONJ patients, who were treated with L-PRF [15]. The same group, conducted another study using L-PRF, where they applied it alone and in combination with BMP-2, and observed complete or partial resolution in 88% and 96.7% patients, respectively [31]. On the other hand, although the first study to use L-PRF in patients using denosumab was unable to achieve complete resolution, the authors reported relatively favorable outcomes with partial healing and stabilization of disease progression [32]. In a recent review, it was reported that L-PRF might have a potential in improving healing as an adjuvant therapy for MRONJ [33]. Another review also concluded that the application of APCs may be helpful in the treatment and prevention of MRONJ, because of their local immunomodulatory properties and possible promotion of angiogenesis and tissue healing by platelet factors [34]. Conversely, in a more recent paper by Escobedo et al., it was concluded that there were no sufficient scientific data to support the use of APCs for the treatment of established MRONJ lesions [35]. Despite extensive research on the role of APCs in MRONJ management, the experimental data seem to remain controversial due to high risk of bias of the studies and the need for further investigation still exists.

Even though the current knowledge recommends stage-specific therapeutic strategies, more studies favoring operative options are being published continuously [36]. In the present study, of the 13 patients with stage II and III MRONJ lesions, nine had complete healing while one had delayed resolution of symptoms and three remained unresolved. A possible explanation for these promising results could be attributed to the large quantity of leukocytes and platelets embedded in a fibrin matrix and gradual release of growth factors and leukocytes migration, as previously reported in various studies [37–39]. When interpreted together with these findings, the authors of this study believe that its encouraging outcomes endorse the use of L-PRF as an adjunct to surgical treatment of MRONJ.

### Study limitations

Main limitations of the present study are the limited sample size and the lack of a control group. Also, the findings need to be carefully assessed due to limitations inherent to the retrospective nature of the study.

## Conclusion

The use of L-PRF may be a favorable adjunctive option in the treatment of MRONJ due to its positive effects on tissue repair, ease of application, minimally invasive and cost-effective character and autogenous nature. Our findings encouraging the use of L-PRF may provide a basis for future research and aid in reaching a consensus on the management of MRONJ. These results, however, need to be confirmed in large, randomized, prospective trials to confirm the true value of this therapeutic option.

**Table 1:** Overview of the patients included in the study

Pt. no.	Gender	Age	Primary disease	BP used	Route	Location	treatment	Secondary intervention	Follow up (months)	Overall outcome
1	F	62	Breast cancer	Zoledronate	IV	Mand	SPO L-PRF	Yes—operated multiple times	27	Incomplete healing—exposed bone after 4 operations. No infection.
2	F	54	Breast cancer	Zoledronate	IV	Mand	SPO L-PRF	No	14	Complete healing
3	M	80	Prostate cancer	Zoledronate	IV	Mand	SPO L-PRF	No	13	Complete healing
4	M	62	Prostate cancer	Zoledronate	IV	Mand	MR L-PRF	No	12	Complete healing
5	M	74	Multiple myeloma	Zoledronate	IV	Mand	MR L-PRF	No	12	Complete healing
6	F	77	Osteoporosis	Alendronate	Oral	Mand	SPO L-PRF	No	14	Complete healing
7	F	79	Prostate cancer	Zoledronate	IV	Mand	MR L-PRF	No	12	Complete healing
8	M	81	Prostate cancer	Zoledronate	IV	Max	SPO L-PRF	No	13	Complete healing
9	F	78	Osteoporosis	Zoledronate	IV	Mand	SPO L-PRF	No	15	Complete healing
10	F	54	Breast cancer	Zoledronate	IV	Max	SPO L-PRF	Yes—OAF treated with flap surgery.	79	Incomplete healing—Partially resolved at 22 months of follow up. No infection.
11	M	74	Prostate cancer	Zoledronate	IV	Mand	SPO L-PRF	Yes, secondary intervention for sequestrectomy	13	Resolved after second intervention
12	F	84	Osteoporosis	Ibandronate, Zoledronate, Denosumab	Oral, IV, IV	Mand	Curettage L-PRF	Yes - operated multiple times.	24	Chronic fistula and pain were eliminated after third intervention.
13	M	82	Prostate cancer	Zoledronate	IV	Mand	SPO L-PRF	No	13	Complete healing

Pt. No., patient number; BP, bisphosphonate; F, female; M, male; IV, intravenous; Mand, mandible; Max, maxilla; SPO, sequestrectomy with peripheral osteotomy; L-PRF, leukocyte- and platelet-rich fibrin; OAF, oroantral fistula

## Data Availability

The datasets analysed during the current study are available from the corresponding author on reasonable request.

## Abbreviations

L-PRF: Leukocyte- and platelet-rich fibrin
MRONJ: Medication-related osteonecrosis of the jaws
AAOMS: The American Association of Oral and Maxillofacial Surgeons
SPO: Sequestrectomy with peripheral ostectomy
BP: Bisphosphonate
BRONJ: Bisphosphonate-related osteonecrosis of the jaws
BMP-2: Bone morphogenic protein-2
APCs: Autologous platelet concentrates
VEGF: Vascular endothelial growth factor
TGF-β1: Transforming growth factor-β1
TGF-β2: Transforming growth factor-β2
IGF: Insulin–like growth factor
PDGF: Platelet-derived growth factor
